# Ravinder Lal Kapur, M.D. (1938–2006)

**DOI:** 10.4103/0973-1229.32290

**Published:** 2007

**Authors:** Lord Crowther, Ajai Singh

**Affiliations:** Inaugural speech as Chancellor of the Open University, 1969; Editor, Mens Sana Monographs, 25 Nov 2006

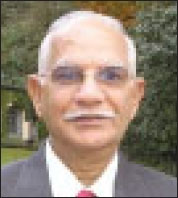



It has been said that there are two aspects of education, both necessary. One regards the individual human mind more as a vessel, of varying capacity, into which is to be poured as much as it will hold of the knowledge and experience by which human society lives and moves. This is the Martha of education and we shall have plenty of these tasks to perform. But the Mary regards the human mind more as a fire that has to be set alight and blown with the divine afflatus. That also we take as our ambition.

## Dr. R. L. Kapur Passes Away

I have to record with profound sorrow the sad demise of Prof R.L Kapur, eminent psychiatrist, thinker and mentor, who was also on the Board of Reviewers of the *Mens Sana Monographs*.

Apart from his high intellectual level, *avant-garde* thought processes and inclinations, and a strong commitment to the furtherance of Psychiatry as a Social discipline (See Obit, p231), what many may not know was his equally strong commitment to learning Hindustani Classical Music. He started late, but his enthusiasm compensated in ample measure for the late start.

One of the fond memories I have is when Dr. Wig, Dr Kapur and I met at Agra last year, and Dr Wig raised the following point over cocktails:

‘One of the prominent Hindi lyricists of today was asked - Which Hindi song is perfect from the point of lyrics. And he answered:

Ye raat ye chandni phir kahan

Sun ja dil ki daastan….’

And while Dr Wig explained why the lyrics were so good, Dr Kapur, holding my hand, just burst forth into the song. I was amazed at his exuberance, and joined in, and so did Dr. Wig.

And, glass in hand, the three of us sang all the three stanzas while the rest were slightly flummoxed as to what was going on.

I met him just a month ago at the Indian Psychiatric Society, South Zone Conference, where he was the guest speaker on Sat, and I was to speak on Sun. And again in the evening we spent a few moments together where he shared with me his experiences of meeting the reclusive Yogis in the Himalayas. He said they may appear reticent but are not really so to the earnest seeker. At least he never had a difficulty communicating with them.

I asked him, ‘So, you go the Himalayas to meet them?’

And he said matter of factly, ‘It's not difficult for you once you have decided. And it's difficult for them to refuse when your sincerity of purpose is obvious’.

And as I allowed the words to sink in, I realised:

1. His was a life led according to objectives decided by the self rather than circumstances.
2. What better example of sincerity of purpose than a life long pursuit of knowledge that was exemplified by his own life?

If Psychiatry retains its unique charm and charisma, it is in no small measure due to the ‘impress’ of souls such as Prof Kapur.

Blessed are those who were taught by him. And blessed are those who came in his contact.

Such souls do not expire. They endure through the institutions they build/work with, and the lives they touch.

